# Prenatal vitamin utilization and its determinants among pregnant women in south Gondar zone: multicenter cross-sectional study

**DOI:** 10.3389/fgwh.2024.1474928

**Published:** 2025-01-06

**Authors:** Begizew Yimenu Mekuriaw, Dagne Addisu, Wassie Yazie Ferede, Fillorenes Ayalew Sisay, Assefa kebie Mitiku, Tegegne Wale Belachew, Tigist Seid Yimer, Habtie Bantider Wubet, Selamawit Girma Tadesse, Negesse Zurbachew Gobezie, Alemie Fentie Mebratie, Moges Kefale Alachew, Temesgen Dessie Mengistu, Yonas Zenebe Yiregu, Rahel Birhanu Arage, Anteneh Mengist Dessie, Fikadu Geremew Gebeyehu, Geremew Bishaw Mekonen, Habtam Desse Alemayehu, Abeba Belay Ayalew, Yitayal Ayalew Goshu, Besfat Berihun Erega

**Affiliations:** ^1^ Department of Midwifery, College of Health Sciences, Debre Tabor University, Debre Tabor, Ethiopia; ^2^ Department of Anesthesia, College of Health Sciences, Debre Tabor University, Debre Tabor, Ethiopia; ^3^ Department of Medical Laboratory, College of Sciences, Debre Tabor University, Debre Tabor, Ethiopia; ^4^ Department of Public Health, College of Health Science, Debre Tabor University, Debre Tabor, Ethiopia; ^5^ Department of Environmental Health, Debre Tabor Health Science Collage, Debre Tabor, Ethiopia; ^6^ Department of Midwifery, College of Health Sciences, Debre Markos University, Debre Markos, Ethiopia

**Keywords:** prenatal, multivitamins, pregnancy, preconception, supplementation

## Abstract

**Background:**

Prenatal vitamin and mineral supplements are commonly advised as clinical practice standard of care. In spite of Ethiopian government focus on maternal nutrition programmes targeting pregnant and lactating women, Micronutrient deficiencies are still quite common and are regarded as a serious public health issue and also little is known regarding utilization and barriers to prenatal vitamin use during pregnancy. This study aimed to assess utilization and associated factors of prenatal vitamins among pregnant women attending antenatal care at public hospitals in the south Gondar zone, 2024

**Methods:**

Multi center crossectional study design was conducted among 416 pregnant women from March 1 to May 30, 2024. Systematic sampling technique was used to select the study participants. Data was collected using Interviewer administered questionnaire. After data, SPSS version 26 software was used for analysis. Factors associated with utilization of prenatal vitamins were identified using bi-variable and multi variable logistic regression models. Statistical significance was declared at 95%CI and *p*-value < 0.05.

**Result:**

In this study, we found that 87.5% (95% CI: 84.31, 90.34) of pregnant women did not use prenatal vitamins. Women not having formal education (AOR = 2.72, 95%CI: 1.44–5.15), being unplanned pregnancy (AOR = 2.58, 95%CI: 1.76–3.78), husband being decision maker in health care (AOR = 1.71, 95%CI:1.09–2.68), having poor knowledge (AOR = 3.27, 95%CI: 1.44–7.42) and unfavorable attitude (AOR = 3.63, 95%CI: 1.61–8.18) on prenatal vitamins were significantly associated with non-users of prenatal vitamins.

**Conclusion:**

The proportion of non-users of prenatal vitamin were higher. Educational level of women, pregnancy plan, decision on health care, knowledge and attitude on prenatal vitamins had statistically significant with utilization of prenatal vitamins. Developing and implementing targeted educational programs to increase awareness about the importance and benefits of prenatal vitamins and encouraging women to take an active role in their healthcare decisions is recommended to improve the utilization of prenatal vitamins.

## Introduction

Pregnancy-related changes in physiology and homeostatic regulation result in an increase in micronutrient requirements (1, 2). Pregnant and childbearing women may not be able to achieve their demands for micronutrients like iron and calcium by food alone, even though increased nutrient consumption should ideally come from food sources (3). Pre-conception folic acid supplementation is recommended by the 2017 Neural Tube Defect Practice Bulletin from the American College of Obstetricians and Gynecologists (ACOG), as it has been demonstrated to lower the incidence and recurrence of neural tube defects (4). However, folic supplementation alone is not enough to cover nutritional gaps. For females of reproductive age, prenatal vitamins are approved as a nutritional supplement during preconception, pregnancy, or breastfeeding (5). Prenatal vitamins are advantageous for both the mother and fetus. The increased micronutrient demands of pregnancy can be addressed, as can dietary inadequacies more generally, with the use of multiple micronutrient supplements (MMS), which comprise 15 crucial vitamins and minerals (6).

Despite the lack of a standard formulation for prenatal vitamins, the United Nations Children's Fund (UNICEF), the United Nations University, and the World Health Organization (WHO) established the International Multiple Micronutrient Antenatal Preparation (UNIMMAP) formulation, which is regarded as the standard for components (7).

The recommended intakes of prenatal vitamins include niacin (18 mg), folic acid (400 µg), copper (2 mg), selenium (65 µg), iodine (150 µg), 30 mg of iron, and 15 mg of zinc for pregnant women. Additional recommended intakes of vitamins B1 (1.4 mg), B2 (1.4 mg), B6 (1.9 mg), B12 (2.6 mg), C (70 mg), D (200 IU), and E (10 mg) are also provided (7–9). The primary source of summary evidence on the effects of MMS was the 2015 Cochrane review, which comprised 17 trials involving 137,791 women. The analysis demonstrated that MMS decreased the risk of low birth weight, being born small for gestational age, and stillbirth (9).

Pregnancy diets high in fish have been demonstrated to be especially advantageous for the Intelligence Quotient (IQ) (intellectual capacity) of the baby (10, 11). However, all pregnant women may not access fish products. One of the key elements is iodine, which has been demonstrated to be beneficial when combined with prenatal vitamins. Children born to iodine-deficient mothers had lower verbal and reading Intelligence Quotient (IQ) scores (12). Recent studies showed that children born from women who had taken prenatal vitamins containing vitamin B12 had improved speech, mathematical abilities and memories (13, 14). In addition, prenatal vitamins have tremendous effect on the reduction of autism spectrum disorder (ASD) in childhood (15, 16). Children whose mothers took prenatal vitamins during the first month of pregnancy had a 14.1% prevalence of autism spectrum disorder, compared to 32.7% in children whose mothers did not take prenatal vitamins during that period (17).

In spite of Ethiopian government focus on maternal nutrition programmes targeting pregnant and lactating women (18), micronutrient deficiencies are still quite common and are regarded as a serious public health issue (19). In 2016, the Ethiopian demographic and health (EDHS) survey found that 23% of pregnant women were anemic (20). Studies showed that 59.9% of pregnant women in Ethiopia were found to be zinc-deficient (21), 23% were vitamin B_12_-deficient (22) and 68.68% were iodine deficient (23).

Despite prenatal vitamin and mineral supplements are commonly advised as clinical practice standard of care, little is known regarding utilization and barriers to prenatal vitamin use during pregnancy in Ethiopia. Therefore, this study aimed to assess utilization and associated factors of prenatal vitamins among pregnant women attending antenatal care at public hospitals in the south Gondar zone.

## Methods and materials

### Study design, setting, and period

A multi-center, institution-based cross-sectional study was conducted among clients attending antenatal care at selected public hospitals in the south Gondar zone, Ethiopia, from March 1 to May 30, 2024. The south Gondar zone is one of the ten administrative zones in the Amhara region, Ethiopia. Debre Tabor town is the capital city of south Gondar zone. The town is located approximately 669 km northwest of Addis Ababa, the capital city of Ethiopia, and 97 km southwest of Bahir Dar, the capital city of the Amhara region. It has an elevation of 2,706 m above sea level. According to the 2022 population projection, Debre Tabor town has a total population of 125,312, with 64,322 males and 60,990 females. Addis Zemen Hospital, Debre Tabor Comprehensive Specialized Hospital, Mekaneyesus Hospital, and Tesfaye Getachew Memorial Hospital were randomly selected among the ten public hospitals in the south Gondar zone.

**Source and study population:** All pregnant women who were attended antenatal care at south Gondar zone public hospitals were the source population for this study. Whereas, selected pregnant women who were attended antenatal care at the selected public hospitals during data collection periods were the study population.

**Inclusion and Exclusion criteria:** The inclusion criteria were pregnant mothers who were attended antenatal care at selected public hospitals during the study period. Women who were seriously ill, unable to hear and/or speak during data collection period were excluded from the study.

### Sample size and sampling procedure

**Figure 1:**
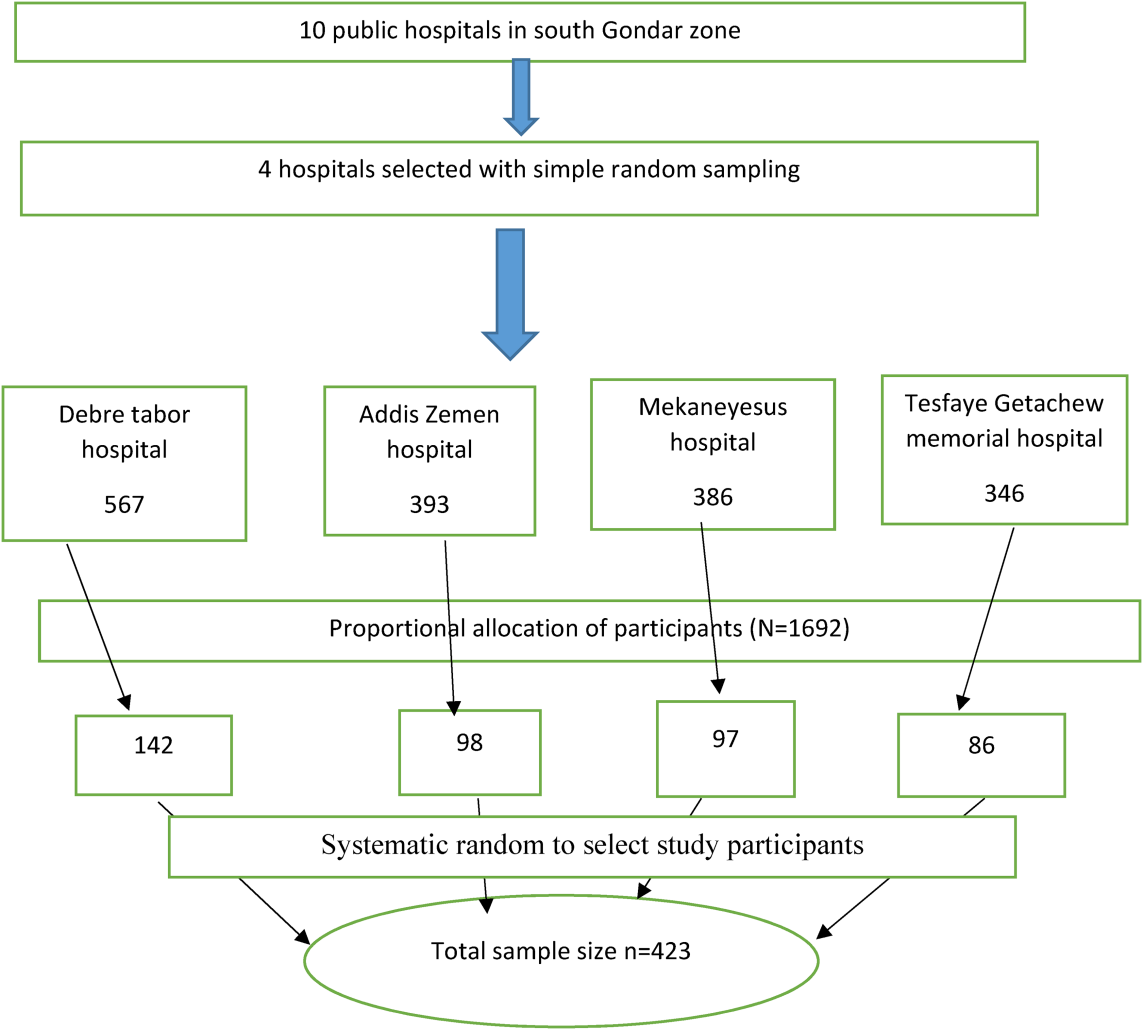
Schematic presentation of the sampling procedure at south Gondar zone public hospitals 2024.

The sample size was determined using a single population proportion formula using a 50% proportion because no previous research had been done in Ethiopia. *P* = 50%, 10% non-response rate, 5% margin of error, and 95% confidence interval. After adding 10% non-response rate, 423 study participants were finally taken into account. Simple random sampling was used to choose the study hospitals. To choose the study subjects, systematic random selection was used. First, we determine the approximate number of women who have used antenatal care within the last three months for the selected facilities. The number of antenatal attendees in each selected hospitals were 567 in Debre Tabor comprehensive specialized hospital, 393 in Addis Zemen hospital, 386 in Mekaneyesus hospital and 346 in Tesfaye Getachew memorial hospital with a total of 1,692. The Kth intervals were calculated by dividing total number of attendees for the total sample size (1,692/423 = 4). The first study participant was selected via lottery method from 1 up to 4 which was 3. Finally, every 3rd pregnant women were interviewed until total sample size was reached (Figure 1).

### Variables of the study

#### Dependent variable

Prenatal vitamin use: yes no

#### Independent variables

### Socio demographic and economic characteristics

Age, religion, educational level of women, educational level husband/partner/, marital status, residency, family size, head of house hold, decision on health care, income generator (the one who earns income in the family).

### Reproductive and health service related factors

Gravidity, parity, pregnancy planned or not, hours to reach health institution, means of transportation, gestational age when antenatal care started.

### Knowledge and attitude

Good knowledge and poor knowledge.

Favorable attitude and unfavorable attitude.

### Operational definitions

**Prenatal vitamin utilization:** it was measured by asking women whether they have used prenatal vitamins before conception or during pregnancy. Then it was considered as users of prenatal vitamin if the response was yes for one of two questions, otherwise non-users.

**Knowledge on prenatal vitamins**: women who scored above the median score (≥3) were classified as having good knowledge otherwise they were considered as poor knowledge (<3).

**Attitude towards prenatal vitamins**: women who scored above the median score (≥2) were classified as having favorable attitude otherwise they were considered as unfavorable attitude (<2).

### Data collection tools and procedures

Validated semi-structured questionnaires from prior studies were used (24–27). The tool was initially prepared in English. To ensure uniformity, this questionnaire was translated into Amharic and then back into English by a different translator. The data were collected by five diploma midwives at the exit of antenatal care service. Two bachelor degree midwives supervised the data collection process, which involved conducting in-person interviews with participants using a semi-structured questionnaire that the interviewer administered. Two days training was given for data collectors and supervisors. Pretest was done on 5% of sample size.

### Quality assurance

To make sure the data was of high quality, a half-day tool discussion with the authors was conducted. To ensure data quality, the supervisors and data collectors underwent comprehensive training on how and what information to collect from the specific data sources. The questionnaires were checked for accuracy and missing information before the data was collected. The principal investigator and supervisors swiftly offered input and checked the completeness of the data each day as it was being collected. The primary investigator cleaned the data before beginning the analysis. A pre-test was conducted 1 week prior to the start of the actual data collection at Megbaru Kebede memorial hospital. Subsequently, the questionnaire was assessed for its length, lucidity, and comprehensiveness, and any requisite modifications were implemented based on the findings.

### Data processing and analysis

Data entry and coding were done using EpiData version 3.1 software. The entered data were exported into the Statistical Package for Social Science (SPSS) version 26 software for further analysis. The analysis process includes several steps. First, basic descriptive statistics such a frequency distribution and percentages were used to describe the respondents' demographic, socioeconomic, and obstetric aspects. Second, a bivariate logistic regression was performed, pairing each independent variable with the outcome variable. If the bivariate test of a variable yielded a *p*-value of less than 0.2, it was considered a possible candidate for the multivariable model. Following the determination of the variables, multivariable analysis was performed. To determine whether the data was appropriate for multiple logistic regression analysis, the Hosmer and Lemeshow goodness-of-fit test was used (*p* = 0.27). Multicolliniarity was checked and there was no Multicolliniarity between independent variables. A multivariable logistic regression model was ultimately developed in order to determine distinct factors of prenatal vitamin utilization. The crude and adjusted odds ratios, as well as the corresponding 95% confidence intervals, were computed to assess the strength of the association. For all two-sided tests, a statistical significance level of *P* < 0.05 was employed.

## Results

### Socio demographic and economic characteristics

In this study, 416 pregnant women participated with response rate of 98.34%. The mean age of the women were 25.8(SD ± 4.62). Among 416 pregnant women, 382 (91.83) of them were orthodox Christians. More than half (53.13%) of principal income generator of the household were husbands (Table 1).

**Table 1:** Socio demographic and economic characteristics of pregnant women attending antenatal care at south Gondar zone public hospitals, 2024 (*N* = 416).

Variables	Categories	Frequencies	Percent
Age	20–24	124	29.81
	25–29	176	42.31
	30–34	116	27.88
Religion	Orthodox	382	91.83
	Muslim	25	6.01
	Protestant	9	2.16
Educational level of women	No formal education	124	29.81
	Primary (1–8)	121	29.10
	Secondary (9–12	107	25.71
	College and above	64	15.38
Occupation	House wife	89	21.39
	Farming	199	47.84
	Merchant	77	18.51
	Civil servant	51	12.26
Marital status	Married	389	93.51
	Single	27	6.49
Educational level of husband	No formal education	104	25.00
	Primary (1–8)	139	33.41
	Secondary (9–12)	78	18.75
	College and above	95	22.84
Residency	Urban	224	53.85
	Rural	192	46.15
Head of household	Husband	383	92.10
	Wife	33	7.90
Decision on health care	Women alone	122	29.34
	Husband alone	125	30.02
	Both	169	40.63
Principal income generator of the household	Women alone	27	6.49
	Husband	221	53.13
	Both	168	40.38
Family size	<3	159	38.22
	3–4	113	27.16
	>4	144	34.62

### Reproductive and health service related factors

More than half (55.04%) of pregnant women were multigravida. Two hundred fifty five (61.25%) of participants reaches to health institution with in 1 h of travel. From the total of study participant, 59.13% of them started antenatal care at second trimester (Table 2).

**Table 2:** Reproductive and health service related factors of pregnant women attending antenatal care at south Gondar zone public hospitals, 2024 (*N* = 416).

Variables	Categories	Frequencies	Percent
Gravidity	Primgravida	187	44.95
	Multigravida	229	55.04
Parity	Nully para	190	45.67
	Primipara	99	23.79
	Multipara	127	30.53
Time taken to reach health institution	Within 1 h	255	61.23
	1–2 h	98	23.56
	>2 h	63	15.14
Means of transportation	By car	202	48.56
	On foot	214	51.44
Pregnancy planned	Yes	157	37.75
	No	259	62.25
Time of booking antenatal care	1st trimester	100	24.04
	2nd trimester	246	59.13
	3rd trimester	70	16.83
Number of ANC contact	<7	377	90.63
	8 or more	39	9.37

### Knowledge and attitude on prenatal vitamin

Among the total respondents, 242 (58.17%) of had no ever heard about prenatal vitamins. Nearly one quarter (25.25%) and 23.56% of study participants had good knowledge and favorable attitude respectively (Table 3).

**Table 3:** Knowledge and attitude on prenatal vitamin of pregnant women attending antenatal care at south Gondar zone public hospitals, 2024 (*N* = 416).

Variables	Categories	Frequencies	Percent
Knowledge			
Have you ever heard about prenatal vitamins?	Yes	174	41.83
	No	242	58.17
Where did you first hear about prenatal vitamins?	Health care providers	111	63.79
	Internet	21	12.07
	Family/friend	42	24.14
Do you know that prenatal vitamin is given for pregnant women?	Yes	72	41.38
	No	36	20.69
	I don't know	66	37.93
Which one of the following is the best time to take prenatal vitamins?	Before conception	41	23.56
	During pregnancy	59	33.91
	During postnatal period	22	12.64
	I don't know	52	29.89
Do you think that pregnant women having healthy diet need prenatal vitamins?	Yes	40	22.99
	No	101	58.05
	I don't know	33	18.96
What are the side effects of prenatal vitamins	Nausea and vomiting	96	55.17
	Constipation	24	13.79
	Stomach pain	46	26.44
	I don't know	8	4.59
How often should prenatal vitamins be taken?	Once a day	108	62.07
	Twice a day	55	31.61
	I don't know	11	6.32
Knowledge	Poor knowledge	311	74.75
	Good knowledge	105	25.25
Attitude			
Do you think that your pregnancy might face vitamin deficiency?	Agree	128	30.77
	Disagree	177	42.55
	Neutral	111	26.68
Do you think that prenatal vitamin supplements really helps you?	Agree	65	15.63
	Disagree	116	27.88
	Neutral	235	56.49
Do you think that prenatal vitamin will improve child's health?	Agree	117	28.13
	Disagree	199	47.83
	Neutral	100	24.04
Do you think that the continuation of the prenatal tablets after Delivery will help you?	Agree	47	11.29
	Disagree	36	8.65
	Neutral	333	80.05
Will you suggest prenatal vitamin supplements to other pregnant women?	Agree	127	30.52
	Disagree	88	21.15
	Neutral	201	48.32
Overall attitude	Unfavorable attitude	318	76.44
	Favorable attitude	98	23.56

### Utilization of prenatal vitamins

**Figure 2:**
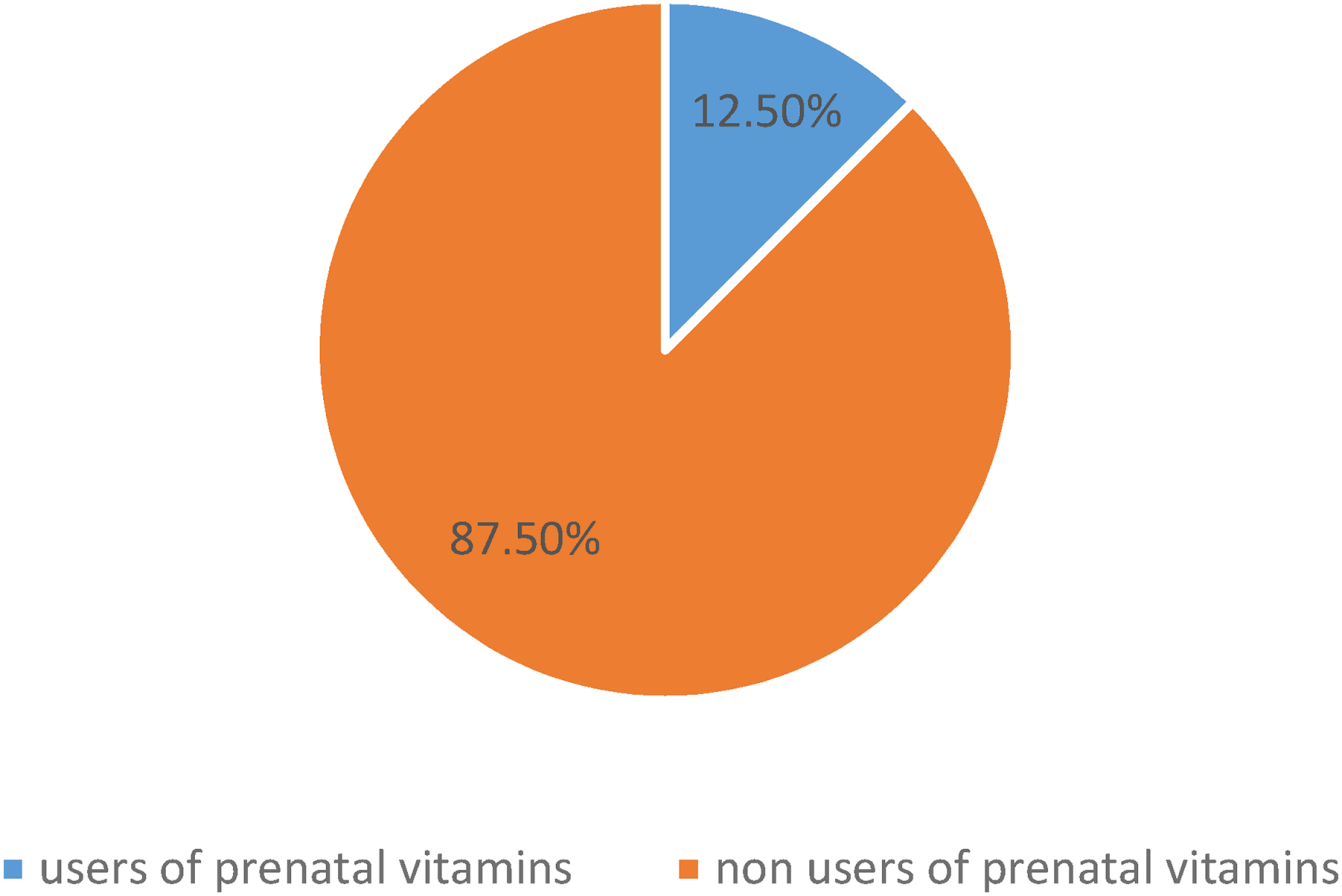
Utilization of prenatal vitamins among pregnant women attending antenatal care at south Gondar zone public hospitals, 2024 (*N* = 416).

In this study, we found that 87.5% (95% CI: 84.31, 90.34) of pregnant women didn't use prenatal vitamins (Figure 2).

### Reasons for not using prenatal vitamins

**Figure 3:**
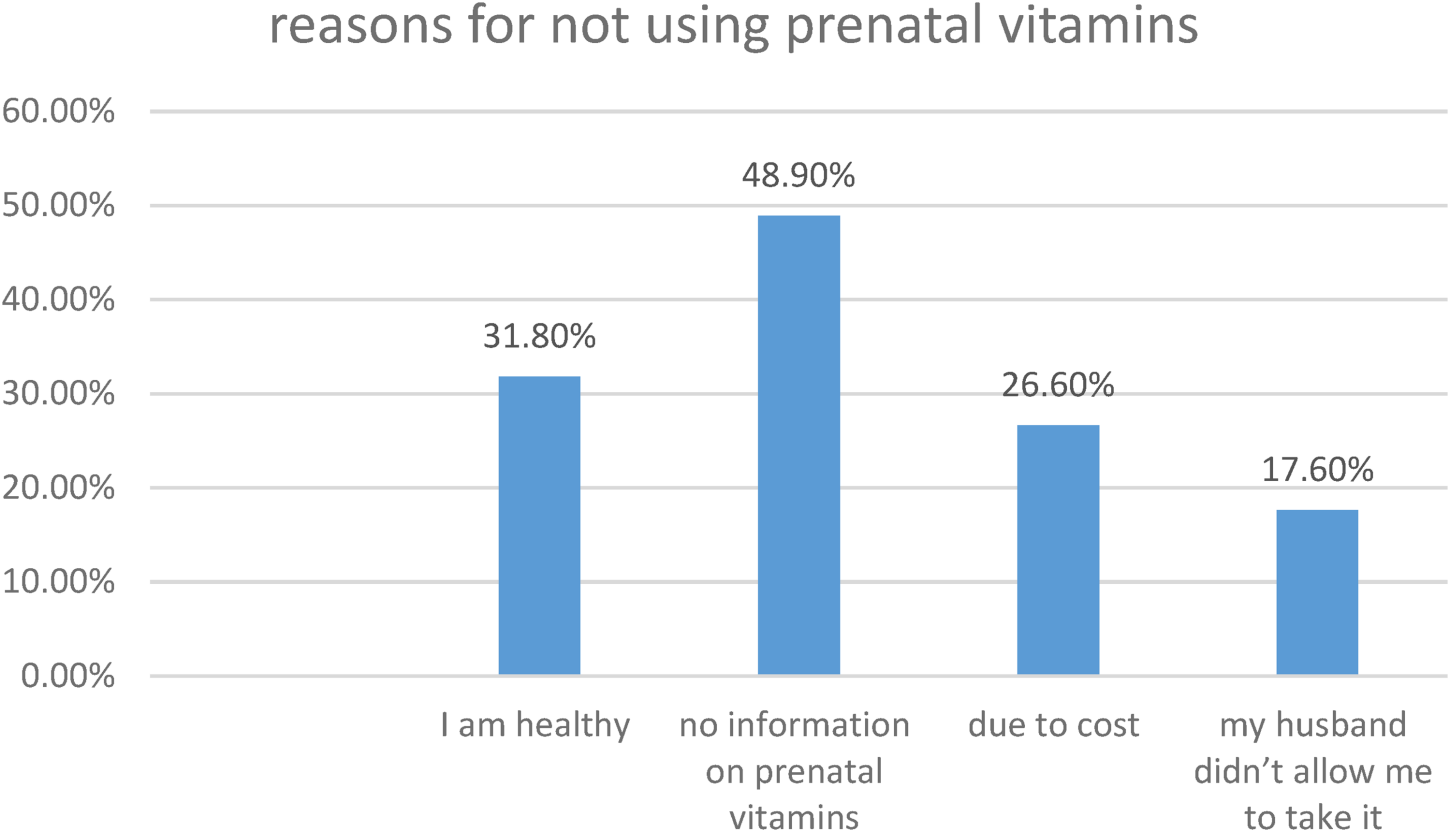
Reasons for non-utilization of prenatal vitamins among pregnant women attending antenatal care at south Gondar zone public hospitals, 2024 (*N* = 416).

From the total study participants, 178(48.9%) of them listed absence of information on prenatal vitamins as the reason for not using it (Figure 3).

### Factors associated with utilization of prenatal vitamins

Age of women, educational status, residency, gravidity, pregnancy planned, decision on health care, knowledge and attitude on prenatal vitamins were candidate variables for multivariable logistic regression analysis.

Women not having formal education were 2.72 times higher to be non-users of prenatal vitamin compared with women whose educational level were college and above (AOR = 2.72, 95%CI: 1.44–5.15). Non-users of prenatal vitamins were 2.58 times higher among unplanned pregnancy compared with planned pregnancy (AOR = 2.58, 95%CI: 1.76–3.78). The odds of being non-users of prenatal vitamins were 1.71 times higher among women whose husband were decision maker in health care compared with women who decide alone (AOR = 1.71, 95%CI:1.09–2.68). Women who had poor knowledge (AOR = 3.27, 95%CI: 1.44–7.42) and unfavorable attitude (AOR = 3.63, 95%CI: 1.61–8.18) on prenatal vitamins were less likely to take it compared with their counter parts (Table 4).

**Table 4:** Bi-variable and multivariable logistic regression analysis of factors associated with utilization of prenatal vitamins among pregnant women attending antenatal care at south Gondar zone public hospitals, 2024 (*N* = 416).

Variables	Utilization of prenatal vitamins		COR(95% CI)	AOR(95% CI)
	Users (52)	Non-users (364)		
Age of women				
20–24	22	102	1.00	1.00
25–29	14	162	2.49 (1.02–6.10)	1.4 (0.84–2.33)
30–34	16	100	1.34 (0.98–1.83)	0.77 (0.52–1.14)
Residency				
Urban	32	192	1.00	1.00
Rural	20	172	1.43 (1.11–1.84)	0.66 (0.38–1.15)
Educational level of women				
No formal education	9	115	3.57 (1.80–7.1)	2.72 (1.44–5.15)*
Primary (1–8)	13	108	2.32 (1.17–4.6)	1.22 (0.96–1.55)
Secondary (9–12)	16	91	1.59 (0.97–2.6)	0.88 (0.74–1.04)
College and above	14	50	1.00	1.00
Gravidity				
Primgravida	30	157	1.00	1.00
Multigravida	22	207	1.79 (0.97–3.30)	0.86 (0.39–1.89)
Pregnancy planned				
Yes	34	123	1.00	1.00
No	18	241	3.7 (2.19–6.25)	2.58 (1.76–3.78)*
Decision on health care				
Women alone	23	99	1.00	1.00
Both	17	152	2.07 (1.29–3.32)	1.31 (0.81–2.11)
Husband alone	12	113	2.2 (1.54–3.14)	1.71 (1.09–2.68)*
Knowledge on prenatal vitamins				
Poor knowledge	23	288	4.78 (2.9–7.87)	3.27 (1.44–7.42)**
Good knowledge	29	76	1.00	1.00
Attitude towards prenatal vitamins				
Poor attitude	21	297	6.54 (3.24–13.2)	3.63 (1.61–8.18)**
Good attitude	31	67	1.00	1.00

COR, crude odd ratio; AOR, adjusted odd ratio; CI, confidence interval; 1.00 = reference.

* *p* value < 0.05.

** *p* value ≤ 0.001.

## Discussion

During pregnancy, prenatal vitamins are essential for maintaining the best possible health for both the mother and the foetus. These dietary supplements are intended to supply essential minerals and vitamins that promote healthy fetal development and to address nutritional deficits. Even with the established advantages of prenatal vitamins, there are still differences in their use across various groups. To improve mother and child health outcomes, it is imperative to design effective public health initiatives that take into account the factors influencing prenatal vitamin use.

In this study, we found that 87.5% of pregnant women were not used prenatal vitamins. It was very higher compared with studies done in Bangladesh 50.23% (25), United States 22% (28) and Florida 47.1% (29). Various factors might contribute to this huge difference. The first reason could be economic factor (30). Since prenatal vitamins are not covered with health insurance or maternal exempted service in Ethiopia, women might not afford for such supplementation rather they prioritize for food and shelter. In addition, civil war between Amhara and Ethiopian military defense force hinders the accessibility of health care (31). Secondly, presence of cultural beliefs which masks modern medical advice might reduce the utilization of prenatal vitamins in developing countries like Ethiopia (32). In some cultures, there may be a preference for natural remedies over synthetic vitamins, affecting utilization.

Educational level of women was one of significant factor affecting prenatal vitamin utilization. Women not having formal education were 2.72 times higher to be non-users of prenatal vitamin compared with women whose educational level were college and above. Similar study done in United States also showed the association between educational level and prenatal vitamin utilization (28). The possible justification could be women not having formal education may not understand the importance of prenatal vitamins because they did not access health information through various channels, such as books and internet (33). Uneducated women might not have better job opportunities and economic stability, which can make it difficult for women to afford prenatal vitamins (34). Another potential reason might be that women not having formal education may have low influence in household decision-making regarding health, including the purchase and use of nutritional supplements (35).

Our study showed that non-users of prenatal vitamins were higher among unplanned pregnancy compared with planned pregnancy. Women who had no plan to be pregnant may not have preconception care (36). If women did not have preconception care, they might not get nutritional information including prenatal vitamins from health care providers which leads to low utilization. Women who are actively trying to conceive usually have a positive outlook towards pregnancy, which can motivate them to take care of their health and adhere to recommended vitamin regimens (37). On the other hand, women who had no plan to conceive may not adhere to nutritional recommendations which makes them for low utilization of prenatal vitamins.

The odds of being non-users of prenatal vitamins were 1.71 times higher among women whose husband were decision maker in health care compared with women who decide alone. This difference might be related with resource allocation. Financial resources may be allocated differently in marriages where men control healthcare decisions. If spouses put other medical costs prior to prenatal vitamins, their wives may not use prenatal vitamins (38). The wife's behaviour may be influenced by her husband's views on maternal health and pregnancy. Lower utilization may result from his lack of support or knowledge regarding the advantages of prenatal vitamins (39).

Knowledge of pregnant women on prenatal vitamins was one of the factor affecting prenatal vitamin utilization. Women who had poor knowledge on prenatal vitamins were less likely to take it compared with their counter parts. It was supported with other study in Florida (29). The reason might be due to absence of information on prenatal vitamins (40). In addition there may be misunderstanding of the role of prenatal vitamins for their baby and themselves that leads to low utilization.

Women with unfavorable attitude towards prenatal vitamins were more likely to be non-users of the supplement compared to those with a positive attitude. This difference may be due to the belief that a balanced diet alone can meet all their nutritional needs, leading them to see no need for additional supplementation.

### Policy and practical implications of the study

The results of this study highlight the need for laws governing public health to encourage pregnant women to take prenatal multivitamins, especially in groups where nutrient deficiencies are more likely to occur. Policy makers ought to think about launching educational initiatives that increase the knowledge of prenatal multivitamins. Incorporating prenatal multivitamin supplements in to current maternal health initiatives can also help guarantee that all women have access to vital nutrients, which will lower the prevalence of unfavorable pregnancy outcomes linked to deficiencies. Healthcare professionals should give special attention to talking about the usage of prenatal multivitamins. This entails clearing up any misunderstandings regarding the advantages of vitamins and minerals and offering precise instructions on the kinds required for optimum pregnancy wellness. Additionally, by collaborating with pharmacies and community health organizations, healthcare systems can make it easier for people to obtain reasonably priced prenatal multivitamins. We can improve maternal care and promote healthier pregnancies by implementing these measures, which will eventually benefit women and their offspring.

### Strength and limitation of the study

Being multi-center is one of the strength of the study since it increases the representativeness of the population. At the start of the interview, participants were assured that their responses would be kept entirely anonymous, which helped to minimize the desirability bias. However, absence of adequate literatures on the area makes it difficult to compare and discuss.

## Conclusion

This study concluded that the proportion of non-users of prenatal vitamin were higher. Educational level of women, pregnancy plan, decision on health care, knowledge and attitude on prenatal vitamins had statistically significant with utilization of prenatal vitamins. Developing and implementing targeted educational programs to increase awareness about the importance and benefits of prenatal vitamins is needed to increase the utilization. These programs can be integrated into existing maternal health services and tailored to different educational levels to ensure accessibility and understanding. In addition, encouraging women to take an active role in their healthcare decisions by providing them with comprehensive information on prenatal vitamins and supporting them in making informed choices is also the pillar area to improve the utilization of prenatal vitamins.

## Data Availability

The raw data supporting the conclusions of this article will be made available by the authors, without undue reservation.
