# Supplementary material for: Prenatal vitamin utilization and its determinants among pregnant women in south Gondar zone: multicenter cross-sectional study
Source: Front Glob Womens Health. 2025 Jan 6;5:1474928. doi: 10.3389/fgwh.2024.1474928 (PMC11744017; doi:10.3389/fgwh.2024.1474928)
Supplement: Supplementary file 1 [file Table1.docx]

| **No** | | **Part I Socio Demographic and economic Characteristics** | **Possible responses** |
| --- | --- | --- | --- |
| 101 | | How old are you? | In year …………………………….. |
| 102 | | Your religion? | 1. Orthodox 2. Muslim 3. Protestant   Others specify……….. |
| 103 | | Your educational level? | 1. No formal education 2. Primary 3. Secondary school 4. Collage and above |
| 104 | | Your occupation? | 1. House wife 2. Farming 3. Merchant 4. Civil servant   Other specify …………. |
| 105 | | Your marital status? | 1. Married 2. Single 3. Divorced 4. Windowed |
| 106 | | Your husband’s educational status? | 1. No formal education 2. Primary 3. Secondary school 4. Collage and above |
| 107 | | Your husband’s occupation? | 1. Farming 2. Merchant 3. Civil servant   Other specify …………. |
| 108 | | Residence area | 1.Urban  2.Rural |
| 109 | | Who is the head of the house hold? | 1. Wife 2. Husband   Others specify …….. |
| 1010 | | Decision on health care | 1. Woman alone 2. Woman and husband 3. Husband alone 4. Other |
| 1011 | | Who is the principal income generator of the household? | 1. Wife 2. Husband 3. Both   Other specify …… |
| 1012 | | How many people live in the house hold? | ……………………… |
| **Part II Reproductive and health service related factors** | | | |
| 201 | | How many times have you been pregnant (gravidity)? | 1. Once 2. 2 up to 5 3. More than 5 |
| 202 | | Parity |  |
| 203 | | How many hours you travel to reach for the health services? | 1. Within 1 hour 2. 1 to 2 hour 3. More than 2 hour   I don’t know |
| 204 | | What’s your means of transportation to reach ANC services? | 1. On foot 2. On the back of the horse/mule 3. By car 4. On animal drawn cart   Other specify |
| 205 | | Would the current pregnancy is planned? | 1. Yes 2. No |
| 206 | | At what GA you started ANC? | ………….. |
| 207 | | How many times did you receive antenatal care during this Pregnancy? |  |
| **Part III :Knowledge on prenatal vitamins** | | | |
| 301 | Have you ever heard about prenatal vitamins? | | - - - 1. Yes       2. No |
| 302 | Where did you first hear about prenatal vitamins? | | a. Healthcare provider  b. Internet  c. Family/Friends  d. Books/Magazines |
| 303 | Do you know that prenatal vitamin is given for pregnant women? | | 1. Yes 2. No 3. I don’t know |
| 304 | Which one of the following is the best time to take prenatal vitamins? | | 1. Before conception 2. During pregnancy 3. During postnatal period 4. I don’t know |
| 305 | Do you think that pregnant women having healthy diet need prenatal vitamins? | | 1. Yes 2. No 3. I don’t know |
| 306 | What are the side effects of prenatal vitamins | | 1. Nausea and vomiting 2. Constipation 3. Stomach pain 4. I don’t know |
| 307 | How often should prenatal vitamins be taken? | | 1. Once a day 2. Twice a day 3. I don’t know |
| Part IV: Attitude toward prenatal vitamins | | | |
| 401 | Do you feel that your pregnancy can lead to vitamin deficiency? | | a. agree  b. disagree  c. neutral |
| 402 | Do you feel prenatal vitamin supplements really helps you? | | a. agree  b. disagree  c. neutral |
| 403 | Do you feel prenatal vitamin will improve child’s health? | | a. agree  b. disagree  c. neutral |
| 404 | Do you feel the continuation of the prenatal tablets after  Delivery will help you? | | a. agree  b. disagree  c. neutral |
| 405 | Will you suggest prenatal vitamin supplements to other pregnant women? | | a. agree  b. disagree  c. neutral |
| Part IV: Utilization of prenatal vitamins | | | |
| 501 | Did you have used prenatal vitamins for current pregnancy? | | 1. Yes 2. No |
| 502 | If yes, when you start | | a. before conception  b. during pregnancy |
| 503 | If no, why you didn’t take prenatal vitamins | | 1. I am healthy 2. I don’t have information 3. Due to cost 4. I am not the decision maker to take it. 5. Others (specify) |
